# Self-reported causes of cancer among 6881 survivors with 6 tumour types: results from the PROFILES registry

**DOI:** 10.1007/s11764-021-00989-w

**Published:** 2021-03-01

**Authors:** Carla Vlooswijk, Olga Husson, Simone Oerlemans, Nicole Ezendam, Dounya Schoormans, Belle de Rooij, Floortje Mols

**Affiliations:** 1 Netherlands Comprehensive Cancer Organisation, Utrecht, The Netherlands; 2 Department of Medical Oncology, The Netherlands Cancer Institute, Amsterdam, The Netherlands; 3 CoRPS - Center of Research on Psychological and Somatic Disorders, Department of Medical and Clinical Psychology, Tilburg University, PO Box 90153, 5000 LE Tilburg, The Netherlands

**Keywords:** Illness perception, PROFILES, Cancer survivors, Causes of cancer, Causal attributions

## Abstract

**Objective:**

Our aim was to describe and compare self-reported causal attributions (interpretations of what caused an illness) among cancer survivors and to assess which sociodemographic and clinical characteristics are associated with them.

**Methods:**

Data from five population-based PROFILES registry samples (i.e. lymphoma (*n* = 993), multiple myeloma (*n* = 156), colorectal (*n* = 3989), thyroid (*n* = 306), endometrial (*n* = 741), prostate cancer (*n* = 696)) were used. Causal attributions were assessed with a single question.

**Results:**

The five most often reported causal attributions combined were unknown (21%), lifestyle (19%), biological (16%), other (14%), and stress (12%). Lymphoma (49%), multiple myeloma (64%), thyroid (55%), and prostate (64%) cancer patients mentioned fixed causes far more often than modifiable or modifiable/fixed. Colorectal (33%, 34%, and 33%) and endometrial (38%, 32%, and 30%) cancer survivors mentioned causes that were fixed, modifiable, or both almost equally often. Colorectal, endometrial, and prostate cancer survivors reported internal causes most often, whereas multiple myeloma survivors more often reported external causes, while lymphoma and thyroid cancer survivors had almost similar rates of internal and external causes. Females, those older, those treated with hormonal therapy, and those diagnosed with prostate cancer were less likely to identify modifiable causes while those diagnosed with stage 2, singles, with ≥2 comorbid conditions, and those with endometrial cancer were more likely to identify modifiable causes.

**Conclusion:**

In conclusion, this study showed that patients report both internal and external causes of their illness and both fixed and modifiable causes. This differsbetween the various cancer types.

**Implications for Cancer Survivors:**

Although the exact cause of cancer in individual patients is often unknown, having a well-informed perception of the modifiable causes of one’s cancer is valuable since it can possibly help survivors with making behavioural adjustments in cases where this is necessary or possible.

**Supplementary Information:**

The online version contains supplementary material available at 10.1007/s11764-021-00989-w.

## Introduction

Although there are known risk factors for certain types of cancer, the causes of cancer in an individual patient are often unknown. This can cause cancer patients to develop their own theories about the cause of their cancer. One possible motive for forming these theories is to make sense of one’s circumstances. In psychology, the process in which individuals use common sense theories to attribute causes to certain events, in an attempt to understand them, is known as attribution theory [1]. Causal attributions are interpretations of what caused the illness [2]. This is part of the common sense self-regulation model of illness, in which patients respond to their symptoms and signs of illness by forming cognitive representations (beliefs about the illness) and emotional reactions of the illness, known as illness perceptions, that lead to coping responses [3, 4].

In general, causal attributions are determined by the extent to which someone sees the cause of their disease as either internal or external, and modifiable or fixed [5]. Patients who attribute causal disease perceptions to external (e.g. environment, chance, or a prior health condition) or fixed (e.g. biological, psychological) causes are more likely to experience less control over their condition and its treatment compared to patients who perceive the cause of their disease as internal (e.g. lifestyle, psychological) or modifiable (e.g. lifestyle, stress). Feeling in control over one’s illness might lead to adjustment of health behaviour [6].

A previous study examining causal attributions among American cancer survivors (*n* = 775) of the 10 most common cancers showed that the most common causal attributions were lifestyle (modifiable), biological (fixed), and environmental (fixed) factors [7]. Cancer type was the only characteristic associated with identifying modifiable causes out of an extensive list of sociodemographic, clinical, and psychosocial characteristics. Therefore, the authors urged the need for additional research in larger populations in order to determine whether other characteristics are associated with modifiable attributions. Therefore, our aim was to study this topic in a larger European population–based sample including both solid and non-solid cancers. More specifically, our goal was to (1) describe self-reported causal attributions of cancer survivors with various cancers in a large (*n* = 6881) Dutch population–based sample and (2) assess which sociodemographic and clinical characteristics are associated with modifiable causal attributions. A clear picture on the modifiable causal attributions cancer survivors have on their disease can teach us whether improvements in information provision regarding this topic are necessary. Being well-informed on the probable modifiable cause of one’s illness can possibly help survivors with making behavioural adjustments in cases where this is necessary or possible. In addition, being well-informed about a fixed cause is important since patients then know that they had no influence on it. Therefore, we performed secondary data analyses on a pooled cohort of existing studies with a similar design among survivors of lymphoma, multiple myeloma, colorectal, thyroid, endometrial, and prostate cancer.

## Methods

### Study design and setting

Data from the PROFILES (‘Patient-Reported Outcomes Following Initial Treatment and Long-term Evaluation of Survivorship’) registry were used for secondary analyses [8]. PROFILES is a registry that facilitates studies examining the physical and psychosocial impact of cancer as well as its treatment. PROFILES includes an extensive web-based component and is combined with the clinical data from the Netherlands Cancer Registry (NCR). The PROFILES registry started its first cohort of cancer survivors in 2008 and is still ongoing, including studies on various cancer types.

### Study population

The current analysis includes six study samples from the PROFILES registry, including patients with lymphoma (including chronic lymphocytic leukaemia (CLL)) and multiple myeloma, colorectal, thyroid, endometrial, and prostate cancer [9–16]. Patients were included between May 2008 and May 2013. In all study samples, participants were included if they were older than 18 years at diagnosis and excluded if they were not able to complete a Dutch questionnaire according to their (former) attending specialist (i.e. severe cognitive impairment, non-native speaker, too ill to participate). Ethical approval was obtained for all study samples separately from the local Dutch certified medical ethics committee Maxima Medical Center (CLL and multiple myeloma, #0734; colorectal, #0822; thyroid, this study was reviewed by the Institutional Review Board as deemed non-human subjects research; endometrial #0822 and #NL33429.008.10; and prostate cancer, #0733).

### Data collection

A detailed description of the data collection procedure has been described previously [8]. In brief, in each study, cancer patients were informed about the study via a letter by their (former) attending specialist. This letter contained either an informed consent form and a paper questionnaire, or a secured link to a web-based informed consent form and online questionnaire. In study samples where the secured link was provided, the patient could return a postcard to request a paper-and-pencil questionnaire, if preferred. All participants included informed consent.

### Study measures

#### Sociodemographic and clinical data

Sociodemographic (i.e. date of birth and sex) and clinical (i.e. cancer type, disease stage, primary treatments received, and date of diagnosis) data were obtained from the NCR. Cancer type was classified according to the third *International Classification of Diseases for Oncology* [17], or cancer stage was classified according to TNM [18] or Ann Arbor Code (Hodgkin lymphoma and non-Hodgkin lymphoma). TNM 5 was used for patients diagnosed from 2002 to 2003, TNM 6 for patients diagnosed from 2003 to 2010, and TNM 7 for patients diagnosed from 2010. For chronic lymphocytic leukaemia and multiple myeloma, stage was not determined nor registered. Primary treatments received were classified into surgery, systemic therapy (chemotherapy, targeted therapy, and immune therapy), hormonal treatment, radiotherapy, and active surveillance/no treatment. Information on educational level (low/middle/high) and marital status (partner/divorced/widowed/alone) were collected in the questionnaire. Survivors were also asked to identify comorbid conditions present in the past 12 months. Comorbidity was classified according to the adapted Self-Administered Comorbidity Questionnaire (SCQ) [19] and categorized into no comorbidity, 1, or 2 or more comorbid conditions.

#### Causal attribution

Causal attribution was assessed with one open-ended item taken from the Dutch version of the Brief Illness Perceptions Questionnaire (BIPQ), an instrument used to assess illness perceptions [20]. We only used the open-ended item that assesses causal beliefs whereby survivors are asked to list the three most important causes for their illness. Our analyses were based upon all three listed causal beliefs combined. Participants’ written responses were coded based on a list of causal attributions derived from the literature [7]. Two authors (CV and OH) coded the listed causes, discussed the coding, and resolved doubtful cases. The responses were condensed into 11 broad categories: lifestyle, biological, environmental, chance/luck, stress, existential, prior health condition, psychological, other, unknown, and missing [7]. The category ‘other’ was chosen if patients wrote down something unclear, unusable, or if they did not understand the question; ‘unknown’ meant that patients themselves indicated that they did not know the cause; and ‘missing’ was specified if they did not answer the question. Each causal attribution was categorized as (a) being either internal (lifestyle, stress, biological, psychological) or external (environmental, chance/luck, existential, prior health condition) to the individual and (b) modifiable (lifestyle and stress) or not modifiable/fixed (biological, environmental, change/luck, existential, prior health condition, and psychological) by an individual (Supplemental Table 1).

### Statistical analyses

Simple descriptive analyses were performed to describe the sociodemographic and clinical characteristics of the sample and to determine the most common causal attributions for the total sample and by cancer type. All variables were described as percentages for categorical data or means and standard deviations for continuous data. Also, the percentage patients who reported only internal, only external, or both internal and external causal attributions and the percentage of patients who reported modifiable, fixed, or both modifiable and fixed causal attributions according to tumour type were shown.

Restricting the total sample to those who identified only fixed or only modifiable causes of their cancer (i.e. excluding those who listed both), we assessed the unadjusted association between demographic and clinical characteristics (including cancer type) and identifying modifiable causal attributions using univariate logistic regression analyses. Backward elimination was used in the multivariate logistic regression; all variables were entered in the model and removed once at a time until all variables in the model were significant. Variables significant at the level of <0.05 were retained in the final multivariate model gender, age at questionnaire, hormonal treatment, stage, marital status, comorbidities, and tumour type. We repeated these analyses for those listing both fixed and modifiable causes of their illness as a sensitivity analyses. Power issues prevented us from performing these analyses separately for each tumour type. Statistical analyses were conducted using SAS version 9.4 (SAS Institute, Cary, NC, 1999) and two-sided *P* values of <0.05 were considered statistically significant.

## Results

### Sociodemographic and clinical characteristics

In total, data of 6881 patients were used (colorectal, *n* = 3989; lymphoma, *n* = 993; multiple myeloma, *n* = 156; thyroid, *n* = 306; endometrial, *n* = 741; and prostate, *n* = 696). Over half of them (54%) were male, mean age was 68, 77% had a partner, 61% had a medium educational level, 45% had 2 or more comorbid conditions, and 50% were diagnosed >3 years ago (Table 1).

**Table 1:** Sociodemographic and clinical data according to tumour type

	Total (*n* = 6881), *n* (%)	Colorectal (*n* = 3989), *n* (%)	Lymphoma (*n* = 993), *n* (%)	Multiple myeloma (*n* = 156), *n* (%)	Thyroid (*n* = 306), *n* (%)	Endometrial (*n* = 741), *n* (%)	Prostate (*n* = 696), *n* (%)
Gender							
Male	3683 (54)	2220 (56)	608 (61)	84 (54)	75 (25)	0 (0)	696 (100)
Female	3198 (46)	1769 (44)	385 (39)	72 (46)	231 (75)	741 (100)	0 (0)
Mean age at questionnaire (SD)	68 (11)	69 (10)	61 (14)	67 (10)	56 (15)	67 (9)	71 (7)
Age at questionnaire							
< 55 years	841 (12)	312 (8)	301 (30)	20 (13)	146 (48)	55 (7)	7 (1)
55–64 years	1677 (24)	926 (23)	238 (24)	42 (27)	77 (25)	260 (35)	134 (19)
65–74 years	2452 (36)	1455 (36)	264 (27)	59 (38)	44 (14)	299 (40)	331 (48)
75+ years	1911 (28)	1296 (32)	190 (19)	35 (22)	39 (13)	127 (17)	224 (32)
Primary treatment							
Surgery	5186 (75)	3946 (99)	0 (0)	0 (0)	302 (99)	741 (100)	197 (28)
Systemic treatment	1999 (29)	1193 (30)	674 (68)	124 (79)	0 (0)	8 (1)	0 (0)
Hormonal treatment	218 (3)	3 (0)	0 (0)	0 (0)	6 (2)	0 (0)	209 (30)
Radiotherapy	2109 (31)	1094 (27)	295 (30)	59 (38)	221( 72)	167 (23)	273 (39)
None/active surveillance	367 (5)	4 (0)	210 (21)	21 (13)	2 (0)	0 (0)	130 (19)
Years since diagnose (mean ± SD)	5 (3)	5 (3)	4 (3)	3 (2)	10 (5)	5 (2)	4 (1)
< 2 years	911 (13)	459 (12)	240 (24)	68 (44)	5 (2)	122 (16)	17 (2)
2–3 years	2526 (37)	1614 (40)	257 (26)	46 (29)	56 (18)	227 (31)	326 (47)
> 3 years	3444 (50)	1916 (48)	496 (50)	42 (27)	245 (80)	392 (53)	353 (51)
Stage							
I	2163 (31)	1072 (27)	231 (23)	NA	172 (56)	686 (93)	2 (0)
II	2336 (34)	1515 (38)	210 (21)	NA	59 (19)	55 (7)	497 (71)
III	1504 (22)	1176 (30)	148 (15)	NA	48 (16)	0 (0)	132 (19)
IV	491 (7)	187 (5)	219 (22)	NA	20 (7)	0 (0)	65 (9)
Missing	387 (6)	39 (1)	185 (19)^b^	NA	7 (2)	0 (0)	0 (0)
Marital status							
Partner	5187 (77)	2963 (76)	772 (79)	119 (77)	238 (78)	515 (72)	580 (85)
Divorced/widowed/alone	1574 (23)	955 (24)	203 (28)	35 (23)	68 (22)	203 (28)	110 (16)
Education level^a^							
Low	1181 (18)	793 (20)	142 (15)	27 (17)	33 (11)	71 (10)	115 (17)
Middle	4087 (61)	2341 (60)	586 (60)	97 (63)	192 (63)	467 (66)	404 (59)
High	1453 (22)	760 (20)	241 (25)	31 (20)	80 (26)	174 (24)	167 (24)
Comorbidities							
0	1592 (25)	911 (25)	292 (32)	27 (19)	75 (25)	122 (18)	165 (25)
1	1922 (30)	1078 (29)	292 (32)	44 (31)	92 (31)	198 (29)	218 (33)
2 or more	2899 (45)	1713 (46)	338 (37)	69 (49)	114 (39)	362 (53)	284 (43)

*NA*, not applicable

^a^Education levels included the following categories: low=no/primary school, middle=lower general secondary education/vocational training, or high=pre-university education/high vocational training/university

^b^Including patients with chronic lymphatic leukaemia

### Most common causal attributions

The 5 most often reported causal attributions were unknown (21%), lifestyle (19%), biological (16%), other (14%), and stress (12%) (Table 2). Those with colorectal cancer reported lifestyle-related factors (24%), biological factors (18%), unknown (17%), stress (13%), and other (11%) most often. Those with lymphoma most often mentioned unknown (28%), other (18%), stress (13%), lifestyle (12%), or chance/luck (10%) as causes of their cancer. Multiple myeloma patients also reported unknown reasons (29%), other (21%), chance/luck (11%), environmental (11%), or biological (10%) as causes. Furthermore, those with thyroid cancer reported unknown (29%), other (23%), biological (14%), chance/luck (13%), and stress (12%) as factors to be the cause of their cancer. Endometrial cancer survivors mentioned lifestyle (16%), unknown (16%), other (13%), biological (12%), and stress (10%) while prostate cancer survivors reported unknown (30%), other (21%), biological (20%), lifestyle (10%), or chance/luck (7%) as the most common causes of their cancer.

**Table 2:** The 3 most important causes of cancer reported by survivors according to tumour type, n(%)

	Total (*n* = 6.881)	Colorectal (*n* = 3989)	Lymphoma (*n* = 993)	Multiple myeloma (*n* = 156)	Thyroid (*n* = 306)	Endometrial (*n* = 741)	Prostate (*n* = 696)
Lifestyle^1,3^	1311 (19)	958 (24)	123 (12)	13 (8)	32 (10)	115 (16)	70 (10)
Alcohol drinking	100 (1)	83 (2)	7 (1)	0 (0)	0 (0)	0 (0)	10 (1)
Smoking/tobacco	219 (3)	160 (4)	26 (3)	1 (1)	7 (2)	7 (1)	18 (3)
Delay in health care	148 (2)	107 (3)	13 (1)	4 (3)	3 (1)	14 (2)	7 (1)
Diet	596 (9)	505 (13)	42 (4)	6 (4)	9 (3)	14 (2)	20 (3)
Use of hormones	38 (1)	1 (0)	3 (0)	0 (0)	2 (1)	29 (4)	3 (0)
Reproductive history	54 (1)	2 (0)	3 (0)	0 (0)	1 (0)	41 (6)	7 (1)
(Multiple) harmful behaviour(s)	292 (4)	221 (6)	31 (3)	3 (2)	7 (2)	8 (1)	22 (3)
Sun exposure	3 (0)	2 (0)	1 (0)	0 (0)	0 (0)	0 (0)	0 (0)
Less activity	113 (2)	106 (3)	4 (0)	0 (0)	1 (0)	0 (0)	2 (0)
Work	90 (1)	59 (1)	24 (2)	1 (1)	3 (1)	3 (0)	0 (0)
Being overweight/obese	82(1)	57 (1)	6 (1)	0 (0)	2 (1)	16 (2)	1 (0)
Biological^1,4^	1084 (16)	699 (18)	94 (9)	16 (10)	43 (14)	90 (12)	142 (20)
Age	157 (2)	71 (2)	8 (1)	3 (2)	2 (1)	10 (1)	63 (9)
Heredity/genetics	979 (14)	657 (16)	86 (9)	14 (9)	41 (13)	82 (11)	99 (14)
Environmental^2,4^	343 (5)	160 (4)	85 (9)	17 (11)	38 (12)	16 (2)	27 (4)
Air pollution	68 (1)	47 (1)	10 (1)	2 (1)	3 (1)	4 (1)	2 (0)
Asbestos	11 (0)	4 (0)	4 (0)	2 (1)	0 (0)	0 (0)	1 (0)
Environment	151 (2)	61 (2)	40 (4)	8 (5)	25 (8)	7 (1)	10 (1)
Household chemicals	8 (0)	3 (0)	3 (0)	0 (0)	1 (0)	1 (0)	0 (0)
Occupational hazards	72 (1)	28 (1)	23 (2)	6 (4)	5 (2)	2 (0)	8 (1)
Second-hand smoke	12 (0)	7 (0)	1 (0)	0 (0)	2 (1)	0 (0)	2 (0)
Toxins	46 (1)	20 (1)	12 (1)	2 (1)	3 (1)	2 (0)	7 (1)
Health care radiation	19 (0)	5 (0)	3 (0)	2 (1)	7 (2)	1 (0)	1 (0)
Chance/luck^2,4^	455 (7)	213 (5)	100 (10)	17 (11)	40 (13)	38 (5)	47 (7)
Stress^1,3^	815 (12)	534 (13)	128 (13)	13 (8)	36 (12)	75 (10)	29 (4)
Existential^2,4^	19 (0)	7 (0)	8 (1)	0 (0)	1 (0)	2 (0)	1 (0)
Prior health condition^2,4^	393 (6)	219 (5)	72 (7)	14 (9)	17 (6)	50 (7)	21 (3)
Infection/bacteria/virus	27 (0)	5 (0)	14 (1)	1 (1)	0 (0)	4 (1)	3 (0)
Previous medical condition	292 (4)	182 (5)	45 (5)	10 (6)	11 (0)	29 (4)	15 (2)
Trauma injury	4 (0)	2 (0)	0 (0)	1 (1)	1 (0)	0 (0)	0 (0)
Medication	45 (1)	22 (1)	9 (1)	1 (1)	2 (1)	9 (1)	2 (0)
Previous cancer	37 (1)	12 (0)	10 (1)	1 (1)	1 (1)	10 (1)	3 (4)
Medical treatment	22 (0)	13 (0)	1 (0)	1 (1)	4 (1)	2 (0)	1 (0)
Psychological^1,4^	63 (1)	34 (1)	7 (1)	0 (0)	7 (2)	10 (1)	5 (1)
Other	958 (14)	430 (11)	183 (18)	32 (21)	70 (23)	98 (13)	145 (21)
Unknown	1419 (21)	685 (17)	275 (28)	46 (29)	88 (29)	117 (16)	208 (30)
Missing	4949 (72)	2837 (71)	701 (71)	112 (72)	194 (63)	601 (81)	504 (72)

Patients were asked to fill in the 3 most important causes of their cancer, but a large majority filled out only 1 or 2. Therefore, the percentages do not sum up to 100%

^1^Internal (lifestyle, stress, biological, psychological) [7]

^2^External (environmental, chance/luck, existential, prior health condition) [7]

^3^Modifiable (lifestyle and stress) [7]

^4^Fixed (biological, environmental, change/luck, existential, prior health condition, and psychological) [7]

**Fig. 1:**
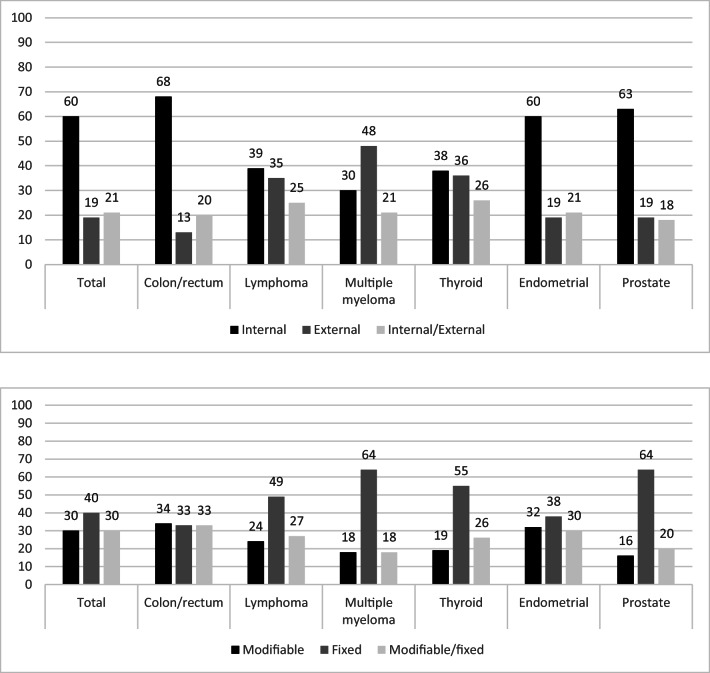
**a** Percentage of only internal (*n* = 1749), only external (*n* = 549), or both (*n* = 597) causal attributions according to tumour type. Internal: lifestyle, biological, stress, psychological [7]; external: environmental, chance/luck, existential, prior health condition [7]. **b** Percentage modifiable (*n* = 864), fixed (*n* = 1161), or both (*n* = 870) causal attributions according to tumour type. Modifiable: lifestyle, stress [7]; fixed: biological, environmental, change/luck, existential, prior health condition, psychological [7]

Colorectal (68%), endometrial (60%), and prostate (63%) cancer survivors reported internal causes of their cancer (i.e. lifestyle, biological, psychological, stress) most often, whereas multiple myeloma survivors more often (48%) reported external causes (i.e. environmental, chance/luck, existential, prior health condition), while lymphoma and thyroid cancer survivors had almost similar rates of internal and external causes (Fig. 1a).

Lymphoma (49%), multiple myeloma (64%), thyroid (55%), and prostate (64%) cancer patients mentioned fixed causes (i.e. biological, environmental, change/luck, existential, prior health condition, psychological) of their cancer far more often than modifiable causes or a combination of modifiable/fixed (Fig. 1b). Colorectal (33%, 34%, and 33%) and endometrial (38%, 32%, and 30%) cancer survivors mentioned causes that were fixed, modifiable (i.e. lifestyle or stress), or modifiable/fixed almost equally.

### Associations with sociodemographic and clinical characteristics

In our sample, 2025 survivors (29%) listed only modifiable (*n* = 864; 43%) or only fixed (*n* = 1161; (57%)) causes of their cancer. Gender, surgery, hormonal treatment, active surveillance/no treatment, stage, marital status, comorbid conditions, and cancer type were associated with reporting modifiable cause of cancer in univariate analyses (Table 3). In multivariate analyses, sex, age, hormonal treatment, stage, marital status, comorbid conditions, and tumour type were associated with reporting modifiable cause of cancer (Table 3). Females, those with a higher age, those treated with hormonal therapy, and those diagnosed with prostate cancer were less likely to identify modifiable causes while those diagnosed with stage 2 disease, those without a partner, with 2 or more comorbid conditions, and those with endometrial cancer were more likely to identify modifiable causes.

**Table 3:** Univariate and multivariate associations between survivors’ sociodemographic and clinical characteristics and reporting only modifiable illness attributions

	*N*	Reported modifiable cause (%)	Univariate, odds ratio (CI)	Univariate, *p* value	Multivariable, odds ratio (CI)	Multivariable, *p* value
Gender						
Male	1105	494 (45)	Ref			
Female	920	370 (40)	0.83 (0.70–0.99)	0.042	0.60 (0.49–0.75)	<.0001
Age at questionnaire (mean, SD)	864	65.3 (11)	0.99 (0.99–1.01)	0.785	0.99 (0.98–0.99)	0.007
Primary treatment						
Surgery	1516	715 (47)	0.46 (0.37–0.58)	<.0001		
Systemic treatment	605	258 (43)	1.00 (0.83–1.21)	0.99		
Hormonal treatment	60	18 (30)	1.76 (1.01–3.09)	0.047	0.47 (0.23–0.98)	0.044
Radiotherapy	626	266 (42)	1.01 (0.84–1.22)	0.915		
No treatment/active surveillance	120	34 (28)	1.95 (1.30–2.932)	0.001		
Years since diagnosis (mean, SD)	864	5.0 (3)	1.01 (0.98–1.04)	0.652		
Stage						
I	618	269 (44)	Ref		Ref	
II	689	300 (44)	1.06 (0.83–1.35)	0.061	1.23 (0.95–1.60)	0.028
III	446	200 (45)	1.06 (0.83–1.35)	0.026	1.01 (0.76–1.34)	0.799
IV	147	64 (44)	1.00 (0.70–1.44)	0.263	1.20 (0.79–1.81)	0.200
Marital status						
Partner	1548	636 (41)	Ref		Ref	
No partner	465	222 (48)	1.31 (1.06–1.61)	0.011	1.41 (1.12–1.77)	0.003
Education level^a^						
Low	258	108 (42)	Ref			
Middle	1210	538 (44)	1.11 (0.85–1.46)	0.101		
High	536	211 (39)	0.90 (0.67–1.22)	0.164		
Comorbidities						
0	513	195 (38)	Ref		Ref	
1	583	239 (41)	1.13 (0.89–1.45)	0.758	1.29 (0.99–1.67)	0.577
2 or more	829	378 (46)	1.37 (1.09–1.71)	0.007	1.47 (1.15–1.88)	0.010
Tumour type						
Colorectal	1189	603 (51)	Ref		Ref	
Lymphoma	294	98 (33)	0.49 (0.37–0.64)	0.636	0.47 (0.34–0.65)	0.958
Multiple myeloma	54	12 (22)	0.28 (0.15–0.53)	0.074	0.41 (0.16–1.05)	0.721
Thyroid	105	27 (26)	0.34 (0.21–0.53)	0.123	0.33 (0.20–0.55)	0.121
Endometrial	182	84 (46)	0.83 (0.61–1.14)	<.0001	1.11 (0.76–1.62)	<.0001
Prostate	201	40 (20)	0.24 (0.17–0.35)	0.0001	0.15 (0.09–0.25)	<.0001

^a^Education levels included the following categories: low=no/primary school, middle=lower general secondary education/vocational training, or high=pre-university education/high vocational training/university

This table only includes patients who mentioned modifiable illness attributions (i.e. lifestyle and stress)

Sensitivity analyses including those with only modifiable causes of cancer in comparison with those reporting both modifiable and fixed causes (*n* = 870) showed no significant differences in the abovementioned results (data not shown).

## Discussion

Overall, the most common causal attributions were unknown (21%), lifestyle (19%), biological (16%), other (14%), and stress (12%). A previous smaller study on causal attributions among American survivors with partly overlapping cancers showed a similar percentage of ‘unknown’ (21.8%). Of those who did provide a causal attribution, results were quite similar as well with the three most common causal attributions being lifestyle (39%), biological (including hereditary; 35%), and environmental (24%) [7]. The fact that the categories ‘unknown’ and ‘chance/luck’ were reported by patients most often is in accordance with reality since most often, we indeed do not know the exact cause of someone’s cancer and it is often related to chance or luck. If we look more closely at the separate cancer groups, we see that realistic causal attributions are often mentioned (biological, lifestyle, chance/luck). However, most cancers have multiple causes, not all causes are clear at the moment, and the exact cause of cancer in individual patients is often unknown. However, a small part of patients also mentioned various unrealistic ideas about the cause of their cancer and information provision in general can thus be improved. Unrealistic perceptions might prevent patients from making behavioural adjustments in cases where this is necessary or possible.

Internal causes of cancer were most often mentioned by colorectal (68%), endometrial (60%), and prostate (63%) cancer survivors, while multiple myeloma survivors (48%) reported external causes most often, and lymphoma and thyroid cancer survivors mentioned internal and external causes equally often. This is quite realistic in the case of colorectal [21], endometrial [22], and thyroid [23–27] cancer and for lymphoma [28, 29]. However, although the literature on the causes of multiple myeloma is still emergent, we do know that risk factors are not only external (e.g. exposures to chemicals or pesticides, overweight and obesity, patterns of alcohol intake [30]) but also internal, in contrast to what survivors in our study reported. The relative lack of knowledge on the causes of multiple myeloma in the scientific literature is thus likely noticeable in the information provision towards patients.

Fixed causes of cancer were most often mentioned by lymphoma (49%), multiple myeloma (64%), thyroid (55%), and prostate (64%) cancer patients whereas colorectal (33% and 34%) and endometrial (38% and 32%) cancer survivors mentioned both fixed and modifiable respectively. This is not surprising since well-known modifiable risk factors (e.g. obesity) exist for colorectal [31] and endometrial [32] cancer, whereas modifiable risk factors for lymphoma, multiple myeloma, thyroid, and prostate cancer are less clear. Those mentioning a modifiable cause of cancer might be more likely to take action in order to adjust this cause when possible (e.g. lifestyle). However, we do know from the literature that threatening illness perceptions, including causal attributions, are not related to a healthier lifestyle [33].

A meta-analysis of studies on illness perceptions showed that illness perceptions predicted outcomes in various patient groups [34]. Illness perceptions can also influence the process of coping [35, 36] and adherence [37, 38] in a wide range of diseases. Interventions aimed at changing illness perceptions seem to be effective. Two brief in-hospital intervention studies, in which patients had individual in-hospital meetings with a psychologist, changed the perceptions of myocardial infarction patients and this resulted in a faster return to work in the intervention group [3, 39]. Interventions that specifically change causal attributions of cancer survivors in order to improve patient-reported outcomes, health care utilizations, or other outcomes are, is to our knowledge, not currently available.

### Study limitations

The present study has some limitations that are worth mentioning. First, the present study is based upon data from a selection of PROFILES studies, although data collection methods were similar. Also, the included cancer types (colorectal, endometrial, thyroid, and prostate cancer; multiple myeloma; and lymphoma) do not fully represent all cancer survivors. Finally, causal attributions were assessed with a single item from the BIPQ. Therefore, we only have information on the type of casual attribution of patients. Qualitative research is needed to acquire information on *why* people think something caused their cancer.

Despite these limitations, the present study provides an important contribution to the current literature on the importance of causal attributions of cancer survivors. Having a well-informed perception of the cause of one’s cancer can probably help with making behavioural adjustments in cases where this is necessary or possible. Since our results are based on several large population-based studies with high response rates including survivors with various cancer diagnoses, and including both short- and long-term survivors, extrapolating these results to the larger population of cancer survivors seems justified.

### Clinical implications

In conclusion, this study showed that patients report both internal and external causes of their illness and both fixed and modifiable causes. This differs between the various cancer types included in this study. Although it is almost impossible to know the exact cause of someone’s cancer, having a well-informed perception of the cause of one’s cancer might help with making behavioural adjustments in cases where this is necessary or possible. Future studies should investigate whether unrealistic causal attributions of cancer survivors can be altered or prevented. In addition, they should investigate what effects these changed attributions have on behavioural changes, patient-reported outcomes, health care utilizations, or other outcomes.

### Supplementary information

ESM 1 (DOCX 14 kb)

## Data Availability

The data that support the findings of this study are available on request from the corresponding author. The data are not publicly available due to privacy or ethical restrictions.

## Abbreviations

BIPQ: Brief Illness Perceptions Questionnaire
NCR: Netherlands Cancer Registry
PROFILES: Patient-Reported Outcomes Following Initial Treatment and Long-term Evaluation of Survivorship
SCQ: Self-Administered Comorbidity Questionnaire
